# Cross-modal Association between Auditory and Visuospatial Information in Mandarin Tone Perception in Noise by Native and Non-native Perceivers

**DOI:** 10.3389/fpsyg.2017.02051

**Published:** 2017-12-04

**Authors:** Beverly Hannah, Yue Wang, Allard Jongman, Joan A. Sereno, Jiguo Cao, Yunlong Nie

**Affiliations:** ^1^Language and Brain Lab, Department of Linguistics, Simon Fraser University, Burnaby, BC, Canada; ^2^Phonetics and Psycholinguistics Laboratory, Department of Linguistics, University of Kansas, Lawrence, KS, United States; ^3^Department of Statistics and Actuarial Science, Simon Fraser University, Burnaby, BC, Canada

**Keywords:** cross-modal association, gesture, audio-visual, tone perception, Mandarin, English

## Abstract

Speech perception involves multiple input modalities. Research has indicated that perceivers establish cross-modal associations between auditory and visuospatial events to aid perception. Such intermodal relations can be particularly beneficial for speech development and learning, where infants and non-native perceivers need additional resources to acquire and process new sounds. This study examines how facial articulatory cues and co-speech hand gestures mimicking pitch contours in space affect non-native Mandarin tone perception. Native English as well as Mandarin perceivers identified tones embedded in noise with either congruent or incongruent Auditory-Facial (AF) and Auditory-FacialGestural (AFG) inputs. Native Mandarin results showed the expected ceiling-level performance in the congruent AF and AFG conditions. In the incongruent conditions, while AF identification was primarily auditory-based, AFG identification was partially based on gestures, demonstrating the use of gestures as valid cues in tone identification. The English perceivers’ performance was poor in the congruent AF condition, but improved significantly in AFG. While the incongruent AF identification showed some reliance on facial information, incongruent AFG identification relied more on gestural than auditory-facial information. These results indicate positive effects of facial and especially gestural input on non-native tone perception, suggesting that cross-modal (visuospatial) resources can be recruited to aid auditory perception when phonetic demands are high. The current findings may inform patterns of tone acquisition and development, suggesting how multi-modal speech enhancement principles may be applied to facilitate speech learning.

## Introduction

From infancy onward, language users are continually tasked with solving the cross-modal binding problem in processing multi-sensory linguistic stimuli (Spence, 2011). According to the theory of embodied-grounded cognition, language processing involves embodying a pre-stored linguistic representation grounded across sensory and motor systems based on communicative contexts (Barsalou, 2008; Borghi et al., 2013). This account predicts joint contributions of sensory and motor systems to perception, indicating that sensory signals from multiple input modalities for the same event must be matched for processing. This requires the perceiver to determine that the inputs are cross-modally associated in a meaningful manner (e.g., spatiotemporally correspondent, semantically congruent) (Ernst and Bülthoff, 2004; Fujisaki and Nishida, 2007; Spence, 2011).

In face-to-face speech interactions, such cross-modal connections may involve integrating auditory acoustic information and visual articulatory configurations (which produce the acoustic output) (Jongman et al., 2003; Munhall et al., 2004), or associating speech with manual gestures (which share similar semiotic representations) (McNeill, 2005; McCafferty, 2006; Kelly et al., 2017). Cross-modal binding may also take the form of cross-sensory equivalence, such as the metaphoric use of spatial stimuli (e.g., high or low) to equate speech information (e.g., high or low pitch) (Bernstein and Edelstein, 1971; Marks, 1987). Moreover, if multi-sensory binding requires reference to pre-stored linguistic representations (Barsalou, 2008), perceivers of different languages may engage in different integration patterns depending on which sensory input they give more weight to (Ernst, 2007; Parise and Spence, 2009; Wang et al., 2009; Hirata and Kelly, 2010).

The present study explores the issues regarding multi-sensory integration and equivalence in cross-modal association by examining native and non-native Mandarin lexical tone perception with auditory and facial articulatory input as well as hand gestures tracing tonal contours. The goal is to test the strength of the cross-modal association between acoustic information and visuospatial information for tone, to determine whether facial and gestural inputs bias tone perception in a linguistically significant manner and how linguistic experience affects such cross-modal association.

### Facial Cues for Speech

Regarding cross-modal association between auditory and visual facial information, previous research has observed auditory-face vowel binding in infants as young as 2 months of age (Kuhl and Meltzoff, 1982; Patterson and Werker, 2003). During critical times for learning their native language (L1), infants have been found to shift their gaze patterns from looking primarily at the speaker’s eyes to the speaker’s mouth (Lewkowicz and Hansen-Tift, 2012). Similarly, for adults, complementary visual articulatory information can be recruited to improve signal quality when listening comprehension conditions are less than ideal, such as when learning a second language (L2), listening to non-native speakers, or perceiving speech in noise (Summerfield, 1983; Jongman et al., 2003; Hazan et al., 2006; Wang et al., 2008, 2009; Kawase et al., 2014; Kim and Davis, 2014).

However, the extent to which facial cues enhance speech perception may vary depending on multiple factors, including the nature of the speech input, the intelligibility of the auditory cue, information value and saliency of the visual cue, the linguistic experience of perceivers, and processing load (Hardison, 1999; Hazan et al., 2005). For example, in terms of the nature of the speech input, when conversing in noisy environments or when repeatedly asked for clarification, speakers may modify their speaking style to produce clear speech with exaggerated visual cues for jaw displacement, lip stretching, lip rounding, and duration (Kim and Davis, 2014; Tang et al., 2015). These modifications have been shown to enhance speech intelligibility (Sumby and Pollack, 1954; Gagné et al., 2002). Research has further revealed that the facial areas that may provide the most visual benefit depend on the type of information sought, such as the eyebrows and upper part of the face for prosody or the lower part of the face for word-level information (Cavé et al., 1995; Lansing and McConkie, 1999; Swerts and Krahmer, 2008). Moreover, when the intelligibility of the auditory cue is degraded, such as in a noisy environment, perceivers rely more on visual cues to enhance speech perception (Sumby and Pollack, 1954; Summerfield, 1979; Hazan et al., 2010). With respect to visual saliency, it has been found that the more visually salient sounds (e.g., labial consonants) offer greater perceptual gains than less visually salient sounds (e.g., alveolar and velar consonants) (McGurk and MacDonald, 1976; Hazan et al., 2006, 2010). Perceiver experience may also affect the extent of visual benefit, with non-native perceivers (compared to native perceivers) showing increased reliance on visual information and enhanced visual benefit, such as in the perception of audiovisually mismatched McGurk tokens (e.g., auditory /ba/ with visual /ga/) produced by non-native speakers (Sekiyama and Tohkura, 1993; Chen and Hazan, 2007). However, the addition of visual information may be inhibitory, particularly when task demands are high. For example, unfamiliar L2 visual cues may cause difficulty in non-native perception (Wang et al., 2009; Kawase et al., 2014). Likewise, perception may be impeded by excessive processing load, when task and attentional demands exceed perceptual load capacities (Lavie, 1995; Alsius et al., 2005).

### Mapping Facial Cues to Tonal Distinctions

Of particular relevance for the present study is the contribution of facial cues to the perception of lexical tone, a prosodic feature used to distinguish word meanings. While there is consensus among previous studies with respect to how visual cues benefit segmental speech perception, findings on visual effects at the prosodic level have been inconclusive.

Some studies have indeed revealed facial effects on the perception of prosody (Lansing and McConkie, 1999; Chen and Massaro, 2008). Head, neck, and eyebrows are shown to produce visible cues to tone perception (Burnham et al., 2001a,b; Mixdorff et al., 2005; Chen and Massaro, 2008), as well as perception of intonation, stress, and contrastive focus (Yehia et al., 2002; Munhall et al., 2004; Kim et al., 2014). For instance, back and forth head movement may accompany a change in tone contours (Attina et al., 2010), dropping of head or chin may signal a dipping tone (Chen and Massaro, 2008), and eyebrow raising may be related to increases in vocal pitch (Huron and Shanahan, 2013).

However, it has been further revealed that these visual cues are not necessarily used until they are brought to perceivers’ attention (Chen and Massaro, 2008), or when listening conditions are challenging, such as in noisy backgrounds (Mixdorff et al., 2008), or in a non-native language (Burnham et al., 2006; Smith and Burnham, 2012). Furthermore, research has not revealed any robust articultory correlates to tone perception in terms of mouth movements. Although tone production results show different mouth movement patterns for different tones, no perception data are available to validate their roles in intelligibility (Attina et al., 2010). Thus it is not clear whether the facial cues revealed in these studies are articulatorily relevant cues to signal tonal category distinctions or are simply attention-grabbing cues. This is presumably because tonal production does not rely on vocal tract configurations and may thus be less visually salient (and more auditorily dominant). However, it may also be the case that the upward or downward articulatory movements of the head or eyebrows (e.g., head dipping, eyebrow raising or lowering) found in previous research (e.g., Kim et al., 2014) are associated with the raising or lowering of pitch (Huron and Shanahan, 2013). Further research is needed to determine whether facial cues (along with acoustic cues) modulate tonal distinctions in a linguistically significant manner.

### Co-speech Gestures

Co-speech gestures may be helpful to both the process of producing speech and perceiving speech (see Hostetter, 2011 for a review). The production of co-speech manual gestures can aid speakers in the management of processing load, where gestures in physical space can act as additional visuospatial working memory resources, thereby relieving the speech modality of having to shoulder the entirety of the semiotic burden of communication (Goldin-Meadow et al., 2001; Wesp et al., 2001). Research has also shown that gesturing during speech can improve fluency, especially when retrieving lexical items that contain spatial content (Rauscher et al., 1996).

Speech marked with gestures provides collocutors with concomitant auditory and visual accents, focus cues, and visual representations of the speaker’s message (Alibali et al., 2001; Krahmer and Swerts, 2007). Previous studies have indicated that perceiving gestures with speech can indeed aid speech perception in an L1. For example, beat gestures have been shown to alter the perception of word prominence, providing additional parsing and focus cues for the perceiver (Krahmer and Swerts, 2007; Biau and Soto-Faraco, 2013). Co-speech gestures also enable perceivers to more easily represent visuospatial aspects in speech comprehension (Wu and Coulson, 2007). This bimodality increases the total possible communicative value of an utterance (McNeill, 2000), where a mismatch of speech and gesture can express two beliefs at once, signaling a gap in understanding or a transitional state of learning (Goldin-Meadow et al., 1993).

However, the extent to which gestures may facilitate speech perception in an L2 exhibits more complex patterns. On the positive end, the addition of gestural input has indeed been shown to facilitate lexical access, as well as rhythm and syllabification in L2 learners (Sueyoshi and Hardison, 2005; McCafferty, 2006). Gestures have also been used by L2 instructors to aid sentence comprehension and vocabulary learning (Barnett, 1983; Lazaraton, 2004; Gullberg, 2006; Gluhareva and Prieto, 2016). Gestures are not always beneficial to learning, though, especially when the tasks involve a fine-grained phonetic perceptual judgment. For example, iconic gestures were found to aid word learning only when word pairs containing the target phonemic contrasts were highly dissimilar in their surrounding segmental contexts, or when words were learned in isolation (Kelly et al., 2009; Kelly and Lee, 2012). As well, hand gestures signaling length of sounds were not effective in helping learners to discriminate phonemic vowel durational differences (Hirata and Kelly, 2010; Hirata et al., 2014; Kelly et al., 2014). Together, these findings may suggest that gestures are more effective for enhancing perception of non-native speech in higher (lexical and sentential) linguistic domains when fine-tuned phonemic distinctions are not the primary focus. When faced with highly demanding phonemic tasks, thus imposing high processing load, additional gestural input may be distracting.

### Mapping Gestures to Tonal Distinctions

Although little research has explored the use of gesture in linguistic pitch processing, the cross-modal association between auditory pitch and spatial movement has been well established in the general cognitive domain (Casasanto et al., 2003). Capturing pitch in gestures owes its inspiration to the illustrative aids of musical expression. For example, to create strong audio-spatial connections, early stage learners in the Kodály music education system are encouraged to kinesthetically engage in their experience of music using gestures and physical movements (Houlahan and Tacka, 2008). Music teachers are taught to enhance pitch perception using hand levels and diagrams of melodic contours (Welch, 1985; Apfelstadt, 1988), and young singers are trained to improve their pitch accuracy using such gestures (Liao, 2008; Liao and Davidson, 2016).

Indeed, it has been claimed that pitch is audio-spatial in representation, which implies that pitch perception should inescapably be affected by spatial information (Connell et al., 2013). When participants were asked to represent stimulus sounds gesturally in a three-dimensional space, higher pitch was generally found to correlate with higher elevation in space (Küssner et al., 2014). Moreover, upward and downward hand gestures were shown to bias pitch perception in the direction of the gesture; and such gesture-directed pitch perception bias appeared to be driven by spatial mechanisms rather than verbal labeling strategies, since the bias remained under increased verbal memory load but disappeared under increased spatial memory load (Connell et al., 2013). It has further been found that musicians are more consistent in associating gestures with pitch height and direction in space than non-musicians, indicating that the strength of cross-modal associations can develop with experience (Küssner et al., 2014).

In a linguistic context, gestures have indeed been shown to affect the perception of pitch in a few studies on intonation and lexical tone perception in an L2. Kelly et al. (2017) found that upward or downward hand movement congruous with the direction of the intonational pitch contour (rising or falling, respectively) could facilitate perception of intonation in an L2, whereas incongruous gesture-pitch matching was disruptive. However, the effects of gesture on lexical tone perception and learning are not clear. Morett and Chang (2015) trained English perceivers to learn Mandarin tone words either with or without viewing hand gestures tracing tone contours. The results showed greater post-training improvements for the group who received training with gesture compared to the no-gesture training group. However, this difference only held true for the word-meaning association task, which involved matching the 12 tone words used in training to their respective meanings; whereas for tone identification in both the trained and novel stimuli, the gesture-training group showed no additional benefit over the no-gesture training group. As such, it is not clear whether the facilitative effects exhibited by the gesture-training group could be attributed to effective cross-modal pitch-gesture association or a result of memorization using verbal labeling strategies (cf. Connell et al., 2013, as discussed above), as participants might have memorized the word-meaning associations of the 12 words that appeared in training.

### The Present Study

The empirical findings reviewed above support the predictions of the theory of embodied cognition (Barsalou, 2008; Borghi et al., 2013), in that facial articulatory and co-speech gestural cues can be effectively integrated with auditory-acoustic information to enhance speech perception. However, several issues remain when it comes to cross-modal association in lexical tone processing.

First, regarding the link between auditory and visual information in lexical tone perception, it is not clear the extent to which the facial cues (such as head, eyebrow, and mouth movements) revealed in the previous studies (e.g., Burnham et al., 2001a,b; Mixdorff et al., 2005; Chen and Massaro, 2008) are articulatorily required or spatially relevant cues to signal tonal category distinctions, or attention-grabbing cues, since (unlike segments) tone production is not triggered by vocal tract configurations. Similarly, with respect to equating acoustic and spatial information for tone, previous work has not been able to determine if the facilitative effects of gesture on tone learning are due to effective cross-modal pitch-gesture association or arbitrary mnemonic devices (Morett and Chang, 2015). Moreover, research has not directly compared the relative weighting of multiple inputs in tone perception. The contribution of audio, facial and gestural inputs may be affected by the relative saliency of various input modalities such that more salient cues or combinations of cues are weighted more heavily in perception (Hazan et al., 2006; Chen and Hazan, 2007; Wang et al., 2009). However, perception may also be impeded by excessive processing load when too many input modalities are involved, especially for non-native perceivers facing demanding L2 phonemic tasks (Alsius et al., 2005; Hirata and Kelly, 2010).

An investigation of multi-modal tone perception to fill these gaps in the literature has significant theoretical implications with respect to the extent to which speech processing enjoys shared representations across sensory-motor domains. As discussed above, lexical tone provides a unique testing case for cross-modal binding due to the nature of its articulation (which is independent of vocal tract configurations), acoustics (with its perceived pitch being visuospatial), and linguistic status (being phonemic and difficult for non-tonal perceivers).

The present study thus examines the cross-modal association in the perception of Mandarin tones with Audio-Facial (AF, involving speaker facial movements in tone production) and Audio-FacialGestural (AFG, also involving speaker hand gestures tracing tone contours) input modalities by native (Mandarin) and non-native (English) perceivers. To test cross-modal association, the combination of auditory and visual tone input was manipulated to be either congruent, where the auditory tone and visual tone match (e.g., A-Rising + F-Rising in AF, or A-Rising + FG-Rising in AFG), or incongruent, where the auditory tone and visual tone are mismatched (e.g., A-Rising + F-Falling, or A-Rising + FG-Falling).

In terms of establishing a meaningful cross-modal association, we hypothesize that, for facial effects, if perceivers are able to effectively incorporate facial tonal cues (e.g., eyebrow raising, head dipping) as non-arbitrary articulatorily or spatially relevant cues, they would more accurately identify tones with congruent (than incongruent) audio and facial input. Likewise, for gestural effects, if perceivers are able to establish an acoustic-visuospatial link for pitch, they would more accurately identify tones when gestural input is available, and would be more accurate with congruent (than incongruent) audio and gestural input. However, if such audio-visual links are arbitrary, resulting from attentional or mnemonic strategies, we should instead find that congruent and incongruent input result in equal performance. Moreover, regarding the relative weighting of the different input modalities, we expect better performance and increased visual weighting in the AFG condition than the AF condition, since hand gestures along with facial movements provide additional input resources than facial movements alone. Finally, comparing native and non-native effects, we expect native Mandarin (relative to English) perceivers to rely less on visual information, as they possess firmly established auditory tone categories. In contrast, English perceivers would be more affected by facial and gestural input than Mandarin perceivers, as presumably they need additional resources to process challenging L2 tones. However, the presence of multiple input sources could also be detrimental for non-native perceivers if they increase processing load.

## Materials and Methods

This study was carried out with the approval of the Office of Research Ethics at Simon Fraser University (SFU) with written informed consent from all participants.

### Perceivers

Fifty-two native and non-native Mandarin perceivers participated in the perception experiment. The native perceiver group consisted of 26 native speakers of Mandarin (15 female) born and raised in northern China or Taiwan, aged 19–33 (mean: 24). The non-native perceiver group consisted of 26 native speakers of English (14 female) born and raised in Western Canada or the United States, aged 19–30 (mean: 24), with no prior tone language experience or formal musical training (as per the criteria used in Cooper and Wang, 2012). All perceivers reported normal hearing and normal or corrected-to-normal vision, and no history of speech or language disorders.

### Stimuli

#### Characteristics and Types of Stimuli

Eight monosyllabic Mandarin words [(/j𝜀/ *pinyin*: *ye*, /joυ/ *pinyin*: *you*) × four tones (Level, Rising, Dipping, Falling)] were chosen as the experimental stimuli for this study. Eight additional monosyllabic Mandarin words were used as tone familiarization stimuli (/tw/ *pinyin*: *duo* × four tones) and task familiarization stimuli (/k/ *pinyin*: *ge* × four tones).

**FIGURE 1:**
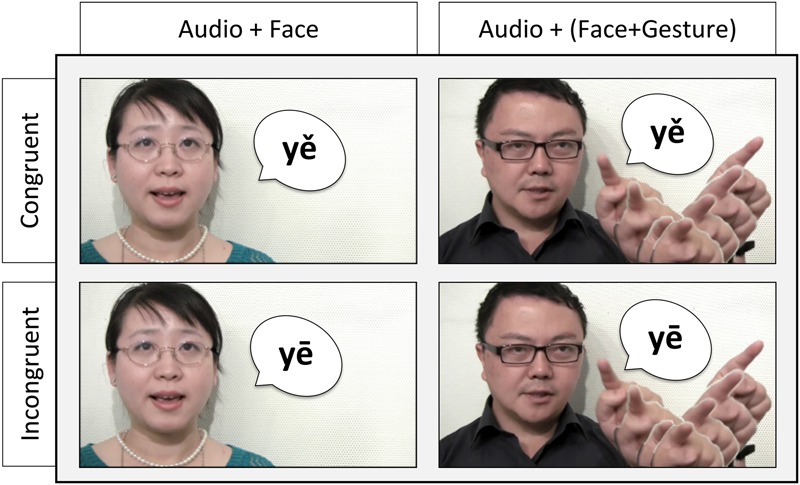
Four types of experimental stimuli, exemplified using the syllable *ye* with Level tone (*yē*) and Dipping tone (*yě*): (1) upper-left panel – congruent Audio and Facial tone input (AF-C): *yě*, (2) lower-left panel – incongruent Audio and Facial tone input (AF-I): audio *yē* + video *yě*, (3) upper-right – congruent Audio and FacialGestural tone input (AFG-C): *yě*, and (4) incongruent Audio and FacialGestural tone input (AFG-I): audio *yē* + video *yě.*

Two modalities were recorded for the target stimuli: Audio + Facial, where the speaker’s facial movements were presented while speaking a corresponding target stimulus; and Audio + FacialGestural, where the speaker made a matched tone contour shaped hand gesture in the space next to their face as indicated by an acetate tone graph on the LCD feedback monitor of the video camera while speaking (e.g., making a high steady left to right hand movement while saying a high-level tone, making a slanted dropping hand movement for a falling tone). The stimuli were further edited to create two incongruent audio-visual stimulus types, where the auditory tone input does not match the facial and gestural tone input (see the section “Stimulus Editing” for details on stimulus editing). Thus, in total, four types of stimuli were developed, as illustrated in **Figure 1**: (1) congruent Audio and Facial tone input (AF-C), (2) incongruent Audio and Facial tone input (AF-I), (3) congruent Audio and FacialGestural tone input (AFG-C), and (4) incongruent Audio and FacialGestural tone input (AFG-I).

#### Speakers and Recording

The experimental stimuli were produced by two (one male, one female) native Mandarin-speaking instructors with experience teaching college-level introductory Mandarin classes. Two additional native Mandarin speakers (one male, one female) produced the audio-only tone familiarization stimuli. The speakers were aged 25–35 and reported no history of speech or language disorders.

Audio-visual recordings were made of the speakers producing the target tokens in citation form in the AF condition in a sound-attenuated booth at the Language and Brain Lab at SFU. The speakers were positioned such that their head, eyebrow, and mouth movements were clearly visible. These facial features were kept neutral except during speech, when facial actions consisted of mouth/jaw opening, lip stretching, and head dipping. Neck bulges and ligament movements were also consistently visible. In the AFG condition, recorded after the AF condition, the same speakers were additionally asked to simultaneously say each token and trace a matching tone contour in the space next to their face as indicated by an acetate graph on the LCD feedback monitor of the video camera. Speakers started with their mouths closed and hands lowered, and returned to the rest position between tokens. The Mandarin characters and Pinyin romanizations were presented to the speakers via PowerPoint slides. Videos were captured on a high definition camcorder (Canon Vixia HF30) at a recording rate of 30 fps. Concurrent high quality audio was recorded using a Shure KSM109 microphone at 48 kHz. All speakers provided written consent to have their images included in publications.

#### Stimulus Editing

The videos were edited using Final Cut Pro X to contain one token per stimulus, with the separately recorded high quality audio replacing the audio track captured by the on-camera microphone, using an automated FFMPEG script.

The intensity of the audio track for each stimulus video was normalized to 65 dB SPL. As the stimuli were derived from the Mandarin perceivers’ native language, tone identification tasks in clear (no-noise) audio would likely result in ceiling performance based exclusively on the audio input for the Mandarin group. In order to improve measurement sensitivity and facilitate the examination of audio-facial and audio-facial-gestural associations, the stimuli were embedded in cafeteria noise following previous research (e.g., Wang et al., 2008, 2009). The signal-to-noise ratio (SNR) was empirically established where Mandarin and English pilot participants were tested on a smaller subset of audio-only stimuli embedded in +10, +5, 0, -5, -10, -12, -15, and -18 dB noise. At -12 dB, the error rate was 15% for the Mandarin group and 54% for the English group. At -15 dB, the tonal information started to be significantly masked by noise, resulting in poorer than chance-level performance (particularly for the English group). On the other hand, at -10 dB, the Mandarin group’s performance was close to ceiling (with less than 10% error rate). Thus, -12 dB was adopted as the optimal SNR that maintained the audibility of the tonal information without completely masking the tone information. Consequently, the 65 dB SPL audio track for each stimulus was embedded in 77 dB SPL cafeteria noise using FFMPEG.

The videos were mirrored horizontally so that the tone contour trace in AFG videos would travel left to right for perceivers during the experiment. AF videos were also mirrored for consistency. Each video was 4 s long to ensure that all the articulatory and gestural movements were captured.

For each modality, syllable and speaker, each auditory tone was paired with a tone-congruent video as well as the three other tone-incongruent videos, producing four tone-congruent pairings (one for each tone) and 12 tone-incongruent pairings (with all the possible audio and visual tone pairings differing in tone, e.g., audio-Level + video-Rising). Thus, for example, a tone-incongruent AFG auditory Level tone, visual Rising tone (AFG-A1V2) stimulus would contain the visual track from the original AFG Rising tone recording paired with the auditory track from the AFG Level tone recording. All videos presented during the experiment were cross-spliced in this manner, including the tone-congruent ones, in order to keep the treatment consistent across all stimuli. To accomplish this, a tone-congruent AFG auditory Level tone, visual Level tone (AFG-A1V1) stimulus would contain the visual track from the original AFG recording paired with the auditory track from the AF recording.

Additionally, to address the potential effects of the durational differences across tones (particularly for the tone-incongruent stimuli) in the audio–video pairings, an auditory duration-modified set of stimuli was created. For each stimulus, syllable onsets and offsets for the audio track of each video were manually marked, and the syllable durations were extracted in Praat (Boersma and Weenink, 2015). For each tone pairing, the durational difference between the original and replacement audio tones was calculated, and a stretch/compression factor was applied to the replacement tone based on the original tone duration. Then the replacement audio file was stretched or compressed accordingly. These duration-modified audio segments were then overlaid onto the original video using Final Cut Pro X, aligning the replacement audio with the syllable onset of the original. Thus, the duration-modified pairings were well matched for duration in the auditory and visual input. However, considering that the stretching or compressing of the audio files may affect the (spectral and temporal) naturalness of tones, the duration-unmodified condition with natural audio was also retained. Both sets of stimuli were presented to perceivers as part of the experiment.

In total, each participant perceived 384 test stimuli (192 AF, 192 AFG) over the course of two sessions. Each of the two modalities consisted of 96 incongruent trials (12 tone pairings × 2 syllables × 2 duration modification conditions × 2 speakers) and 96 congruent trials (4 tone pairings × 2 syllables × 3 repetitions × 2 duration modification conditions × 2 speakers). Moreover, eight additional audio files (4 tones × 1 syllable “*duo*” × 2 speakers) not embedded in noise were included as tone familiarization stimuli, and eight additional tone-congruent, noise-embedded audio–video files in each (AF, AFG) modality (4 tones × 1 syllable “*ge*” × 2 speakers) were prepared as task practice stimuli before the experiment.

All AF and AFG tokens were evaluated by two native Mandarin speakers for accuracy as well as audio–video quality check. The speakers correctly identified the stimuli and rated them as satisfactory exemplars of the intended tones and gestures.

### Procedures

The experiments were conducted in a sound-attenuated perception booth at the Language and Brain Lab at SFU. Stimuli were presented using Paradigm Stimulus Presentation software (Perception Research Systems, 2007) on 15-inch LCD monitors. Video stimuli were presented at 1024 × 576 resolution, and audio was presented using AKG circumaural headphones. Participants were scheduled for two 1-h test sessions, separated by at least 1 h break. They were compensated with their choice of $30 cash or SFU course credits.

Prior to the test sessions, participants were introduced to the Mandarin lexical tone system with the eight tone-familiarization stimuli described above, presented in an audio-only condition with the tone descriptors “Level,” “Rising,” “Dipping,” and “Falling” provided visually on the screen. These descriptors capture height and direction of auditory pitch as well as gestural movements. Participants subsequently practiced identifying these tone stimuli by pressing the corresponding buttons on the keyboard. No feedback was given. The participants were required to perform above chance before continuing. They all met this inclusion criterion and none had to repeat the task (Mandarin group mean: 97.3%, SD: 7.3%; English group mean: 72.0%, SD: 16.4%).

After completion of the tone-familiarization exercise, participants moved on to the test sessions. Each session contained half of the stimulus set and was further divided into two test blocks by modality of presentation: AF and AFG. Each block consisted of four practice trials, followed by 96 experimental trials. The speakers, syllables, tones, tone congruency, and duration modification factors were randomized for presentation within each block. Block presentation was counter-balanced across session and participants. The task in each experimental trial required perceivers to watch and listen to a stimulus video of a speaker producing a target token, and then respond to the question “Which tone did you perceive?” by identifying the tone as Level, Rising, Dipping, or Falling, and pressing the correspondingly labeled button on the keyboard. Perceivers were instructed to respond as quickly as possible after the response screen appeared, and were given a maximum of 4 s to respond.

## Results

### Effects of Audio-visual Congruency

**FIGURE 2:**
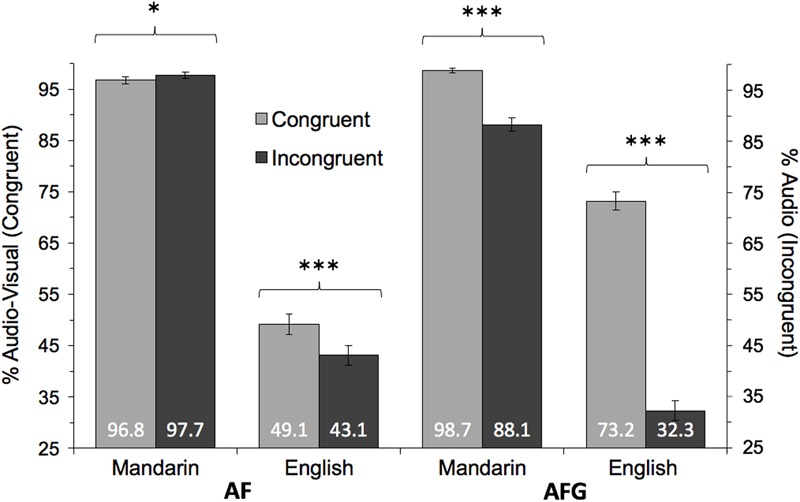
Mean accuracy comparisons between Congruent and Incongruent conditions for each Group (Mandarin, English) and Modality (AF, AFG). Congruent accuracy measured as % responses aligned with audio-visual tone; Incongruent accuracy measured as % responses aligned with auditory tone. Statistically significant Congruency effects indicated at ^∗^*p* < 0.05, ^∗∗^*p* < 0.01, ^∗∗∗^*p* < 0.001. Error bars indicate 95% confidence interval.

First, to evaluate tone perception as a function of the congruency of auditory and visual (facial gestural) input, tone perception accuracy was compared in the congruent and incongruent conditions. The auditory input served as the basis for tone accuracy measurements in the incongruent conditions. **Figure 2** illustrates these congruency comparisons.

The data were submitted to multilevel mixed effect logistic regression with Congruency (Congruent, Incongruent), Group (Mandarin, English), and Modality (AF, AFG) as fixed factors. A random effect was added on the intercept term to account for different perceivers. Factors peripheral to the focus of the study were also adjusted for in the analysis, including Duration modification (Modified, Unmodified), Tone (Level, Rising, Dipping, Falling), Speaker gender (Male, Female), Syllable (*Ye, You*), and Repetition (1, 2, 3). The estimated coefficients, summarized in **Table 1** below, reveal significant main effects of Congruency, Group, and Modality, as well as main effects of Duration modification and Tone. For brevity, only significant interactions involving Congruency, Group, and Modality (the main factors of concern here) are reported. The significant effects involving Duration modification and Tone will be further analyzed in the Sections “Effects of Duration of the Auditory and Visual Input” and “Effects of Individual Tones,” respectively.

**Table 1:** Summary of mixed effect logistic regression model for tone identification accuracy.

Factor	Estimate	Std. error	*z*-value	Wald test *p*-value
(Intercept)	0.92	2.21	0.42	0.677
Congruency	-0.31	0.07	-4.56	<0.001
Group	4.17	0.32	12.98	<0.001
Modality	1.24	0.07	18.70	<0.001
Duration modification	0.19	0.04	4.53	<0.001
Tone	0.05	0.02	2.50	0.012
Speaker gender	-0.01	0.02	-0.50	0.618
Syllable	-0.01	0.04	-0.05	0.958
Repetition	-0.04	0.06	-0.54	0.591
Congruency × Group	0.67	0.19	3.43	0.001
Congruency × Modality	-1.81	0.09	-19.25	<0.001
Congruency × Group × Modality	-1.34	0.29	-4.64	<0.001

As shown in **Table 1**, the logistic regression revealed a significant three-way interaction of Congruency × Group × Modality, as well as two-way interactions of Congruency × Group and Congruency × Modality. To further assess these interactions, likelihood ratio tests for each modality were conducted to determine whether including a Congruency × Group interaction term would improve the model fit compared to a reduced model excluding the interaction term but retaining Congruency, Group, Duration Modification, Tone, Speaker gender, Syllable, and Repetition as factors. Significant interactions of Congruency × Group were found for both AF [χ^2^(1) = 11.50, *p* < 0.001] and AFG [χ^2^(1) = 17.77, *p* < 0.001] modalities. With the same approach, significant Congruency × Modality interactions were observed for the Mandarin [χ^2^(1) = 155.90, *p* < 0.001] and English [χ^2^(1) = 383.64, *p* < 0.001] groups.

These significant interactions motivated further comparisons of Congruency within each Modality and Group, using Wald tests. First, in the AF modality, for Mandarin perceivers, accuracy was unexpectedly higher in the incongruent condition (AF-I Mean: 97.7%, SD: 5.5%) than the congruent condition (AF-C Mean: 96.8%, SD: 4.6%) [AF-C/AF-I = 0.61, CI = (0.42, 0.91), *z* = 2.51, *p* = 0.012], although it should be noted that performance was close to ceiling in both congruency conditions (**Figure 2**). In contrast, for the English group, tone accuracy was significantly higher in AF-C (Mean: 49.1%, SD: 23.5%) than in AF-I (Mean: 43.1%, SD: 20.2%) [AF-C/ AF-I = 1.37, CI = (1.19, 1.58), *z* = -4.53, *p* < 0.001], showing the expected positive effects when congruent auditory and facial information was presented. The positive effects of congruency were also revealed in the AFG modality, where congruent auditory and facial-gestural input (AFG-C) produced higher tone accuracy compared to incongruent input (AFG-I) for both the Mandarin (AFG-C Mean: 98.7%, SD: 2.4%; AFG-I Mean: 88.1%, SD: 22.2%) [AFG-C/AFG-I = 29.88, CI = (17.13, 52.11), *z* = -12.21, *p* < 0.001] and English (AFG-C Mean: 73.2%, SD: 19.0%; AFG-I Mean: 32.3%, SD: 23.1%) [AFG-C/AFG-I = 8.99, CI = (7.61, 10.62), *z* = -26.42, *p* < 0.001] groups.

In sum, the results demonstrate more effective perception with congruent (than incongruent) auditory and visual (facial/gestural) input for both native (Mandarin) and non-native (English) perceivers, with the exception of the Mandarin AF condition where ceiling performance was observed.

### Effects of Input Modality

To determine the extent to which the AFG modality relative to AF affected tone perception for both Mandarin and English perceivers, congruent audio-visual trials were analyzed using logistic regression with Group and Modality as fixed effects, with model adjustments for the same peripheral and random factors as reported in the section “Effects of Audio-visual Congruency.” A significant main effect of Modality was observed across groups, where tone identification was more accurate in AFG (Mean: 86.0%, SD: 18.6%) than in AF (Mean: 72.9%, SD: 29.4%), [AFG/AF = 3.56, CI (3.11, 4.08), *z* = 18.70, *p* < 0.001]. A significant main effect of Group was observed across modalities, with Mandarin perceivers (Mean: 97.8%, SD: 3.8%) outperforming English perceivers (Mean: 61.2%, SD: 24.4%), [Mandarin/English = 48.42, CI (26.05, 90.02), *z* = 12.38, *p* < 0.001]. No significant Modality × Group interaction was observed [χ^2^(1) = 1.51, *p* = 0.22].

**FIGURE 3:**
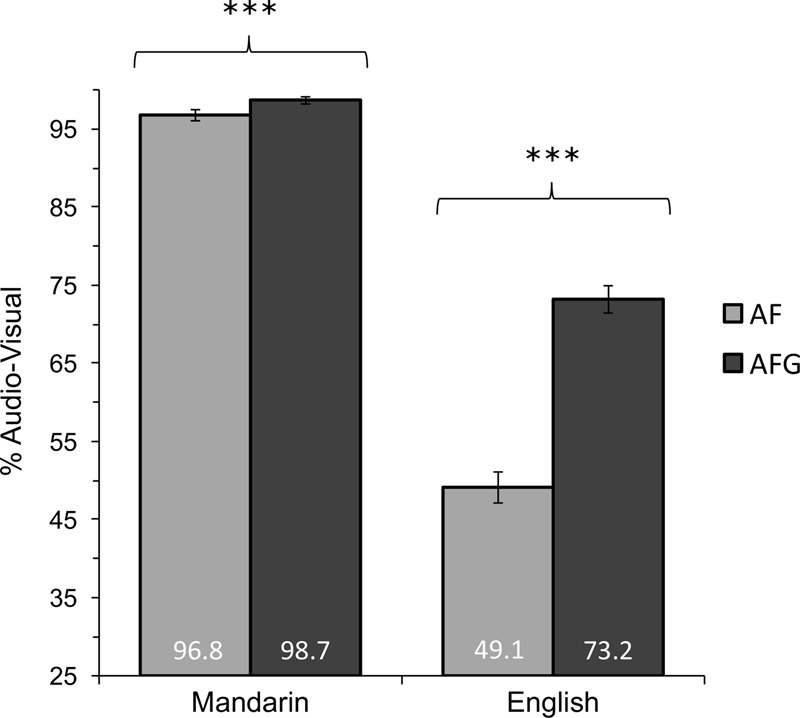
Mean accuracy comparisons for audio-visual tone congruent trials for AF and AFG modalities in the Mandarin and English groups. Statistically significant Modality effects indicated at ^∗^*p* < 0.05, ^∗∗^*p* < 0.01, ^∗∗∗^*p* < 0.001. Error bars indicate 95% confidence interval.

Despite the lack of interaction, the Mandarin perceivers’ near-ceiling performance in both AF and AFG conditions motivated further Wald tests comparing Modality for each Group. The results confirmed that tone accuracy was superior in AFG compared to AF for both native Mandarin perceivers [AFG/AF = 2.78, CI (1.81, 4.27), *z* = 4.77, *p* < 0.001] and English perceivers [AFG/AF = 3.55, CI (3.10, 4.07), *z* = 18.67, *p* < 0.001]. A significant effect of Group for each Modality was observed, with native Mandarin perceivers outperforming English perceivers in both AF [Mandarin/English = 47.94, 95% CI (24.77, 92.75), *z* = 11.50, *p* < 0.001] and AFG [Mandarin/English = 44.25, CI (20.28, 96.54), *z* = 9.50, *p* < 0.001]. **Figure 3** illustrates these differences in performance by Modality and Group.

These results indicate the benefit of gesture, where AFG produced higher tone identification rates compared to AF for both native (Mandarin) and non-native (English) groups. The perceptual benefits of gesture were more pronounced for the non-native group, as the native perceivers achieved very high tone identification accuracy rates with and without gesture, as expected.

### Effects of Perceptual Weighting of Auditory and Visual Input

**FIGURE 4:**
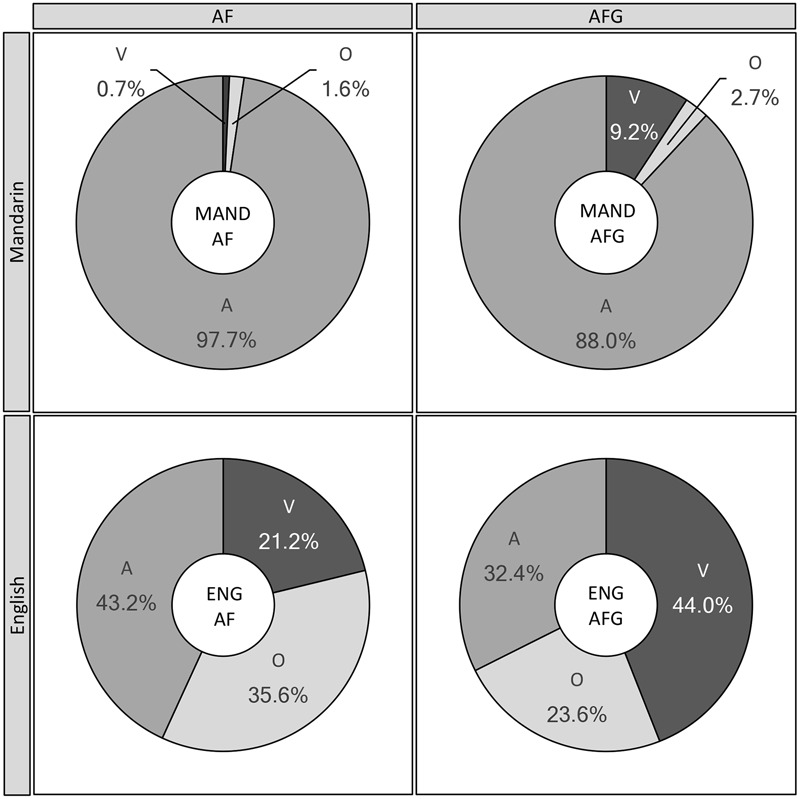
Group (Mandarin, English) and Modality (AF, AFG) comparisons of Incongruent data, classified by Audio, Visual (facial/gestural), or Other response type. A: % correct responses based on audio tone input, V: % correct responses based on visual tone input, O: % other tone responses; MAND: Mandarin group, ENG: English group.

In order to quantify the effects of visual (facial and gestural) relative to auditory information on perception in incongruent AF and AFG conditions, a perceptual weighting analysis in the present section sorted perceiver responses for each token into three categories: correct response based on auditory tone input (A), correct response based on visual tone input (V), or one of the remaining (Other, O) two tones (since participants were given all four tones as response options). For example, in the case of a token consisting of an audio Rising tone cross-spliced with a visual Falling tone, a Rising response would be coded as A, Falling as V, and a Level or Dipping tone response as O. **Figure 4** shows the varying proportion of responses by Modality and Group, categorized as A, V, and O, for the Mandarin and English perceiver groups in AF and AFG conditions.

For each Group and Modality, Friedman’s tests with subsequent Wilcoxon–Nemenyi–McDonald–Thompson *post hoc* tests were conducted to determine the rank order of the participant responses in the A, V, and O response categories. Within-group and modality weighting of A and V proportions were then evaluated using pairwise *t*-tests. These within-group proportions were subsequently submitted to two-sample *t*-tests in order to determine the differences in perceptual weighting between modalities.

For Mandarin perceivers in the AF modality, Friedman’s test results indicated that the A, V, and O response categories were not equally preferred [χ^2^(2) = 11.69, *p* < 0.001]. As expected, the A-based responses were significantly greater than both V-based and O responses (*p*s < 0.001), whereas the latter two categories did not differ significantly (*p* = 0.306). Likewise, in AFG, significant differences among response categories were also observed [χ^2^(2) = 13.06, *p* < 0.001]; responses to A significantly outweighed V, which in turn outweighed O (*p*s < 0.001). However, between-modality comparisons using two sample *t*-tests showed that the A-based responses were significantly greater in AF than in AFG [*t*(25) = 2.49, *p* = 0.020], whereas the V-based responses were significantly greater in AFG than AF [*t*(25) = 2.31 *p* = 0.029].

For the English group, the AF condition also revealed significant differences in audio-visual weighting [χ^2^(2) = 10.61, *p* < 0.001], with *post hoc* tests indicating greater A-based responses over O, which in turn significantly outranked V-based responses (*p*s < 0.001). In the AFG condition, significant differences between category responses were observed as well [χ^2^(2) = 11.69, *p* < 0.001]. However, in contrast to the other results of A-dominant response patterns, with gesture, English perceivers’ responses following the visual input increased to the extent that V exceeded both the A and O categories (*p*s < 0.001); while the latter two did not differ (*p* = 0.079). Comparisons between the AF and AFG modalities showed that English perceivers gave significantly more A responses in AF than AFG [*t*(25) = 4.63, *p* < 0.001], whereas they gave significantly more V responses in AFG than in AF [*t*(25) = 8.67, *p* < 0.001].

**FIGURE 5:**
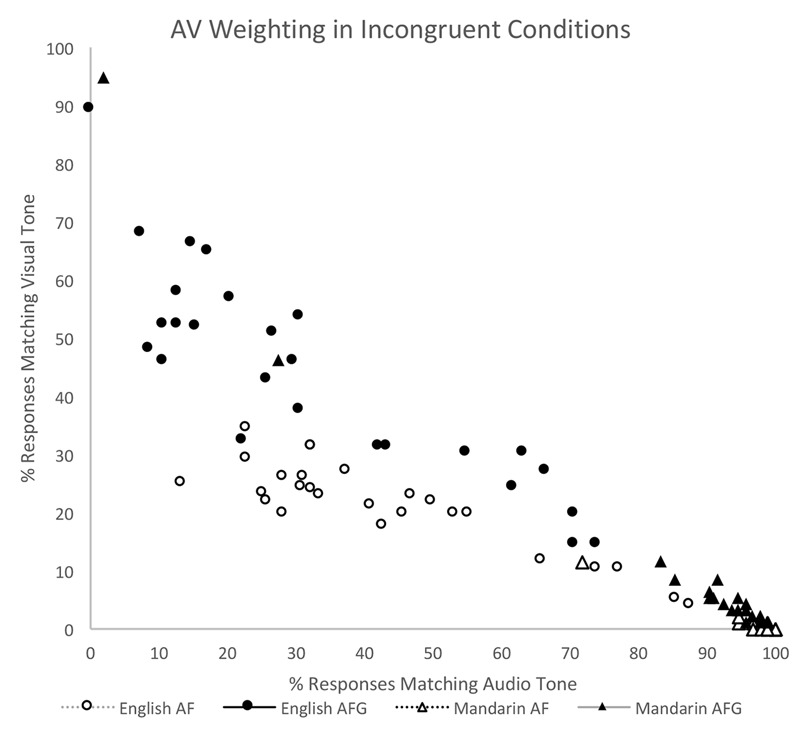
Correlation between auditory and visual response for each Group and Modality pairing (English AF, English AFG, Mandarin AF, Mandarin AFG) in the Incongruent conditions.

Standard deviation results of the incongruent conditions at the group level have thus far suggested an inverse relationship between the variables of auditory (A) and visual (V) response, where A decreases when V increases. Pearson correlation coefficients were calculated to determine the linearity of this relationship in each Group and Modality. Overall, strong negative correlations were found for A and V tone responses for all groups and modalities in the incongruent conditions. In the Mandarin group, significant negative correlations were found for both AF (*r* = -0.97, *n* = 26, *p* < 0.001) and AFG (*r* = -0.98, *n* = 26, *p* < 0.001). Similarly, in the English group, there were significant negative correlations in AF (*r* = -0.88, *n* = 26, *p* < 0.001) as well as in AFG (*r* = -0.88, *n* = 26, *p* < 0.001). **Figure 5** illustrates these relationships by plotting % Audio Tone against % Visual Tone for each Group, Modality, and Participant.

Finally, cross-group comparisons using pairwise *t*-tests showed that both Mandarin [*t*(25) = 2.31, *p* = 0.029] and English perceivers [*t*(25) = 8.67, *p* < 0.001] significantly increased their V weighting from AF to AFG. Within each group, the differences for audio and visual responses between AF and AFG were then calculated for each participant. A two sample *t*-test of the differences revealed that the increase in visual weighting with the inclusion of gesture in the AFG condition was significantly greater for English perceivers (22.8%) than for Mandarin perceivers (8.5%) [*t*(50) = 3.18, *p* = 0.003].

To summarize, the analysis of the incongruent data showed that in stimuli where auditory and visual cues for tone were mismatched, both native (Mandarin) and non-native (English) perceivers increased their visual weighting when highly salient gesture cues were available in the AFG modality (as compared to AF). Furthermore, the non-native group weighted the visual tone even more highly than the auditory tone input when gestures were present.

### Effects of Duration of the Auditory and Visual Input

As discussed in the section “Speakers and Recording,” two sets of stimuli were created for the incongruent stimuli: the duration-modified set with modified audio tone duration to match the duration of the visual tone, and the duration-unmodified set with the natural audio tone duration retained. The main effect of Duration modification in the full model logistic regression in the section “Effects of Audio-visual Congruency” motivated further analysis on the incongruent data to determine if durational congruency affects perception as a function of Modality and Group. A likelihood ratio test between the full model (including all two- and three-way interactions) and the reduced model excluding the interaction term indicated no significant Group × Modality × Duration modification interaction for either the Auditory-based responses [χ^2^(1) = 0.60, *p* = 0.44] or the Visual-based responses [χ^2^(1) = 0.7244, *p* = 0.395]. This result indicated that duration modification affected all groups and modalities in the same way, and therefore no further analysis was undertaken.

### Effects of Individual Tones

The significant main effect of Tone (*p* = 0.012) observed in the full model logistic regression in the section “Effects of Audio-visual Congruency” motivated additional analyses of potential individual tone effects as functions of Modality and Group, for both the audio-visual congruent and incongruent data. First, likelihood ratio tests between the full model and the reduced model, which excluded the interaction term, were used to assess the Tone × Modality × Group interactions. If significant interactions were found, further Friedman’s tests with Wilcoxon–Nemenyi–McDonald–Thompson *post hoc* tests were employed to tease apart the differing effects of individual tones in each modality and for each group. **Figure 5** illustrates individual tone perception in AF and AFG for Mandarin and English perceivers in terms of (a) percent correct identification in the congruent conditions, (b) percentage of responses matching the auditory tone in the incongruent conditions, and (c) percentage of responses that matched visual tone in the incongruent conditions.

**FIGURE 6:**
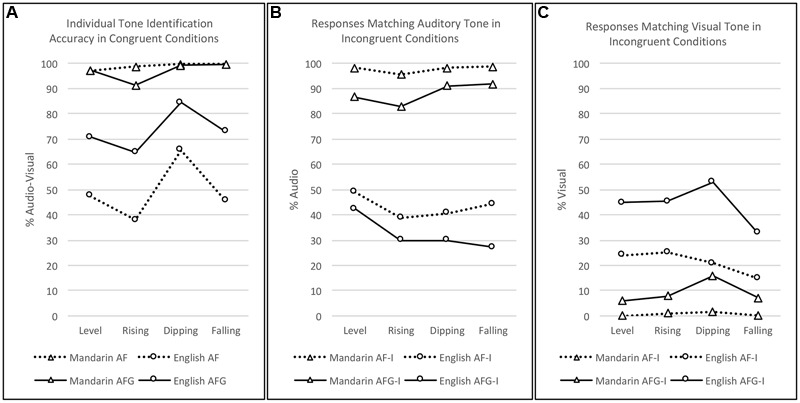
Individual tone (Level, Rising, Dipping, Falling) perception by Group (Mandarin, English) and Modality (AF, AFG). **(A)** Percent correct identification in audio-visual congruent conditions. **(B)** Percentage of responses matching the auditory tone in incongruent conditions. **(C)** Percentage of responses matching the visual tone in incongruent conditions.

Likelihood ratio tests of the Congruent data (**Figure 6A**) revealed a significant interaction of Group × Modality × Tone [χ^2^(3) = 16.561, *p* < 0.001]. Although identification accuracy for each tone was generally very high in the Mandarin perceiver group, Friedman’s test showed significant differences in the AF condition [χ^2^(3) = 32.92, *p* < 0.001], with *post hoc* pairwise comparisons indicating significantly higher accuracy for Rising, Dipping, and Falling tones than Level tone (*p*s < 0.001). A significant tone effect was also observed in AFG [χ^2^(3) = 10.61, *p* = 0.014), showing better perception of Dipping and Falling tones than Rising tone (*p*s = 0.031). A significant effect of tone was also present for the English group in the AF condition [χ^2^(3) = 16.27, *p* = 0.001], with Dipping tone performing better than both Rising and Falling tones (*p*s ≤ 0.017). Likewise, in AFG, the significant tone effect [χ^2^(3) = 13.49, *p* = 0.004] was due to better identification of Dipping than Rising tone (*p* = 0.002).

Analysis of the incongruent data based on audio responses (**Figure 6B**) only revealed a significant Group × Tone interaction [χ^2^(3) = 55.96, *p <* 0.001]. Across modalities, significant differences between tones were found for the Mandarin group [χ^2^(3) = 21.01, *p* < 0.001], but not for the English group [χ^2^(3) = 2.16, *p* = 0.130]. For the Mandarin perceivers, Dipping tone outperformed Rising tone, and Falling tone outperformed both Level and Rising tones (*p*s ≤ 0.024).

The video response analysis of the incongruent data (**Figure 6C**) also only revealed a significant Group × Tone interaction [χ^2^(3) = 38.76, *p* < 0.001]. Across modalities, significant differences between tones were found in both Mandarin [χ^2^(3) = 32.30, *p* < 0.001] and English [χ^2^(3) = 17.63, *p* < 0.001]. For the Mandarin group, Dipping tone responses were greater than all the other tones (*p*s ≤ 0.003). For the English group, Level, Rising, and Dipping tones all outperformed Falling tone (*p*s ≤ 0.020). Moreover, the Modality × Tone interaction was also significant [χ^2^(3) = 25.97, *p* < 0.001]. Across groups, significant differences between tones were found in AF [χ^2^(3) = 3.18, *p* = 0.008] and AFG [χ^2^(3) = 5.69, *p* < 0.001] modalities. In AF, Rising was better than Falling tone (*p* = 0.008); and in AFG, Dipping was better than all the other tones (*p*s < 0.001).

Overall, the most notable result of the individual tone analysis was how frequently Dipping tone was observed to outperform the other tones on the measures that included a visual component, especially in the AFG modality.

### Summary

Taken together, the results show better performance with congruent (than incongruent) auditory and visual (facial/gestural) input across groups, indicating that perceivers are able to make cross-modal associations between acoustic, visual articulatory, and spatial pitch information. And this association was not caused by durational congruency effects. Furthermore, the addition of gestural input increases perceptual accuracy as well as visual weighting over facial input, across all tones and especially for the Dipping tone. The perceptual benefits of visual, particularly gestural, input were more pronounced for the non-native group than the native group.

## Discussion

### Facial Effects

For facial congruency effects, we hypothesized that if perceivers could effectively integrate visual facial cues as articulatorily relevant cues, they would achieve better performance in the audio-facial congruent than incongruent condition. While native Mandarin perceivers did not show the expected congruency effects, presumably due to their ceiling-level performance, the non-native results support our hypothesis in that English perceivers could more accurately identify tones with congruent (than incongruent) auditory and facial information. The English results are consistent with previous findings that facial cues for tone are more likely used by non-native perceivers who find themselves in a challenging non-native phonetic situation, than by native perceivers (Chen and Massaro, 2008; Smith and Burnham, 2012). The fact that the current stimuli were presented in cafeteria noise further added to the challenge. In fact, previous research has shown that, with increased auditory noise, perceivers’ attention increasingly shifts from the eyes to the mouth, indicating a shift to articulatorily relevant cues (Vatikiotis-Bateson et al., 1998). The current non-native performance indeed suggests that the perceivers may have been able to incorporate specific facial movements as articulatorily relevant cues for tone, such as head movements previously found to be tone-relevant (Attina et al., 2010; Kim et al., 2014). Although the current design does not allow us to specify which particular cues contributed to the perceptual patterns, the congruency comparisons indicate that these cues are not just arbitrary cues, since if perceivers did not associate visual cues with auditory cues in a meaningful manner, their perception would not have been different for the congruent and incongruent audio-visual stimuli. Although the results from Mandarin perceivers did not directly support our hypothesis, their high performance in both congruent and incongruent conditions indicates that they possess firmly established auditory tonal categories sufficient for accurate perception, making it less likely for them to be misled by incongruent facial information; as was also shown in previous research (Wang et al., 2008).

The perceivers’ different weighting of audio-visual information is further evidenced by their response patterns in the incongruent condition, where the responses following audio versus visual input were 97.7% and 0.7%, respectively, for Mandarin perceivers, but 43.2% and 21.2%, respectively, for English perceivers. These patterns support our hypothesis that the weighting of auditory and visual information would vary depending on the language background of the perceivers. Consistent with previous findings (Mixdorff et al., 2005), the Mandarin group relied almost exclusively on auditory input, which was sufficient for their accurate perception. The English group, on the other hand, showed a greater reliance on facial information, as was also shown in prior studies (Smith and Burnham, 2012).

Categorizing the current non-native results by individual tone, Dipping tone perception tended to be more accurate than most of the other tones in the audio-facial congruent condition; although in the incongruent condition, the Dipping tone was not more frequently responded to than the other tones following either auditory or visual input. The superior performance in the perception of the audio-facial congruent Dipping tone demonstrates effective integration of auditory and facial cues, indicating the recruitment of valid visual cues in perception. Indeed, in tone perception, Dipping tone is the candidate with the most visually salient features relative to other tones, with a noticeable head dipping or jaw lowering motion corresponding to the articulatory configuration for the turning point of the tone (Chen and Massaro, 2008; Smith and Burnham, 2012).

In face-to-face conversation, there is no reasonable expectation of hearing sounds issuing from a speaker that are in direct opposition to their articulatory configurations, so both the native and non-native performance patterns can be reasonably accounted for in relation to strategically recruiting articulatorily relevant auditory and visual cues in a complementary but integrative manner as needed in perception.

### Gestural Effects

In the present study, both native and non-native perceivers were more accurate in the congruent (than the incongruent) condition, where the hand movements were in the same direction and shape as the tone contours, supporting our hypothesis that perceivers were able to make a cross-modal connection between the tone gesture and the auditory tone. These results compare well with the findings of intonational contrasts that hand-intonation contour congruent gestures resulted in greater accuracy than incongruent gestures (Kelly et al., 2017), and that (non-speech) pitch perception could be swayed upward or downward in the direction of the gesture (Connell et al., 2013; Küssner et al., 2014). The results further confirmed the nature of the gesture-pitch association in the perception of phonemic tone that was not identified in previous research (e.g., Morett and Chang, 2015). That is, the association is due to the audio-spatial nature of pitch (Connell et al., 2013) rather than memorization of arbitrary labeling of a gesture with a specific tone since perceivers’ performance in the congruent and incongruent conditions was different.

In spite of the positive gesture-pitch association across groups, native and non-native perceivers exhibit different audio-visual weighting patterns with the addition of gestural input. Although the Mandarin group showed increased visual weighting as an effect of adding tone gestures to the visual stimuli, their perception appeared to be overwhelmingly audio-based (with the audio vs. visual responses being 88%:9.2% in the incongruent condition), despite the non-optimal listening condition with the tonal stimuli embedded in noise. This pattern is aligned with the previous claim for audio-facial speech perception that the existence of robust auditory categories in native perceivers makes visual input weighted less and thus visual distraction less likely (Mixdorff et al., 2005; Wang et al., 2008). For the English group, however, the proportion of visual-based responses was so large that they out-weighed the proportion of audio-based responses (44.0%:32.4%). These results also agree with the previous findings that non-native (relative to native) perceivers attach greater weight to visual input (Hazan et al., 2006; Wang et al., 2008, 2009). They further provide evidence supporting the claim in the audio-facial domain that the degree of visual weighting positively correlates with the saliency of visual contrasts (Hazan et al., 2006). The individual tone results corroborate the saliency account showing that across groups the Dipping tone tends to be more accurately and frequently responded to, as compared to most of the other tones. This is presumably because the dipping gesture involves a trajectory of a combined falling to rising movement, which is more visually salient than a rising or falling contour alone.

Overall, despite the fact that gestures generally provide redundant cues to concurrent speech (Hostetter, 2011), perceivers are able to cross-modally relate visuospatial gestural tone information to the auditory tone information, and use them effectively when necessary.

### General Discussion

Taken together, the results reveal that cross-modal binding occurred in both AF and AFG conditions. However, a comparison of the two modality conditions showed that perceivers across groups (especially the non-natives) were able to identify tones more accurately and respond to the visual input more frequently when gestural input was available than audio-facial input alone. These results support our hypothesis of an increased visual weighting in the AFG over the AF condition, indicating the effectiveness of hand gestures as an additional and salient input source. The facilitative effects of gestures in non-native tone perception also suggest that this additional channel of input did not make the task more demanding, or increase perceivers’ processing or attention load, as suggested in previous research in non-pitch-related domains (e.g., Alsius et al., 2005; Hirata et al., 2014; Kelly et al., 2014). This result may be due to the audio-spatial nature of pitch (Connell et al., 2013). The fact that cross-modal pitch-gesture binding occurs in the perception of phonemic tone as well as for non-speech and musical pitch (e.g., Liao and Davidson, 2007; Connell et al., 2013; Küssner et al., 2014) indicates that this binding may exist universally in perceivers’ sensory-motor systems and may not need to be learned. Thus, the facilitative effects of the gesture-pitch association can overcome the processing load issue in phonetically demanding contexts.

The current results of an established cross-modal binding both between auditory and facial tone and between auditory and gestural tone suggest shared representations of pitch processing across sensory-motor domains, supporting the theory of embodied-grounded cognition (Barsalou, 2008; Borghi et al., 2013). Specifically, the present AFG results coupled with the previous findings (e.g., Liao and Davidson, 2007; Connell et al., 2013; Küssner et al., 2014) of an acoustic-visuospatial binding for pitch in speech, non-speech, and music imply that pitch representation is not only grounded across sensory-motor systems but also across cognitive domains. This pre-stored representation of pitch information can be recruited to aid perception in a cross-modal and cross-domain fashion. The shared representation account may not only address the gesture-tone link in the AFG results, but also explain the facial articulatory and auditory connection of tone in the AF condition. As discussed previously, the production of tone (unlike that of segments) does not rely on vocal tract configurations, and thus may not necessarily involve visible mouth movements corresponding to specific features of sounds (e.g., lip spreading or rounding for vowels). Nonetheless, previous research has indicated that certain articulatorily relevant cues, such as head dipping, eyebrow raising and lowering (Burnham et al., 2001a,b; Huron and Shanahan, 2013; Kim et al., 2014), do affect pitch perception and production. Relating these results to the account of audio-spatial nature of pitch, it is likely to be the case that the head or eyebrow movements accompanied by tone productions provide spatial equivalence to pitch trajectories, similar to the function of hand gestures. The effective utilization of visual information in the perception of the Dipping tone in both AF and AFG conditions provides a good example illustrating this shared process, in that the dipping pitch trajectory may be visual-spatially realized both as a head dipping and a dipping hand movement. As such, the current audio-visual binding results from facial and gestural domains may be accounted for by common underlying mechanisms in terms of shared acoustic and spatial processing.

## Conclusion

The present findings support our hypothesis that perceivers can make cross-modal, non-arbitrary, audio-spatial correspondences between acoustic and visual tonal cues, and bind them during perception. These findings thus speak to a shared representation of tone across auditory-acoustic, articulatory, and visuospatial domains. On the other hand, the differences in audio-visual weighting between the AF and AFG modalities, and between native and non-native groups, also provide evidence of domain-specific and experience-based influences in tone perception. These patterns advance the previous integrative pitch processing account (Zatorre and Gandour, 2008) by extending the findings to the spatial gestural domain, thus providing insight into how the interaction of multi-sensory and cognitive processing is orchestrated by lower- and higher-level mechanisms.

The theoretical implications of this study point to directions for further research. First, in terms of audio-visual weighting, as the current study involved cafeteria noise intended to increase the level of visual reliance, the results attributed to the enhanced visual effect may not apply to the same stimuli perceived in quiet. Future research could compare how the weighting of cross-modal tonal information varies in different perceptual environments. Furthermore, regarding effects of linguistic experience, the inter-modal relations in tone perception exhibited in the current study may be particularly beneficial for tone language development and learning, where infants and non-native learners need multiple resources to acquire new pitch contrasts. However, research tracing the developmental and learning trajectories is needed to establish how these resources can be utilized effectively at different stages of acquisition. Finally, the current results demonstrate the existence of meaningful cross-modal links for tone without identifying which facial and/or gestural cues contribute to the perception of specific tones. Extended research may seek to quantify the relationship between tone perception and specific visual cues to determine the nature of the shared representation of tone across auditory-acoustic, articulatory, and visuospatial domains. Together, research along these avenues informs how multi-modal speech enhancement principles can be applied to achieve effective human speech interactions.

## Author Contributions

BH mainly contributed to experimental design, stimulus development, data collection, analysis and write-up. YW, AJ, and JS mainly contributed to study planning, experimental design, data analysis and write-up. JC and YN mainly contributed to statistical analysis.

## Conflict of Interest Statement

The authors declare that the research was conducted in the absence of any commercial or financial relationships that could be construed as a potential conflict of interest.
